# The Species-Level Composition of the Fecal *Bifidobacterium* and *Lactobacillus* Genera in Indonesian Children Differs from That of Their Mothers

**DOI:** 10.3390/microorganisms9091995

**Published:** 2021-09-21

**Authors:** Mengfan Ding, Bo Yang, Wei Wei Thwe Khine, Yuan-Kun Lee, Endang Sutriswati Rahayu, R. Paul Ross, Catherine Stanton, Jianxin Zhao, Hao Zhang, Wei Chen

**Affiliations:** 1 State Key Laboratory of Food Science and Technology, Jiangnan University, Wuxi 214122, China; 6160112016@vip.jiangnan.edu.cn (M.D.); zhaojianxin@jiangnan.edu.cn (J.Z.); zhanghao61@jiangnan.edu.cn (H.Z.); chenwei66@jiangnan.edu.cn (W.C.); 2 School of Food Science and Technology, Jiangnan University, Wuxi 214122, China; 3 International Joint Research Center for Probiotics & Gut Health, Jiangnan University, Wuxi 214122, China; micleeyk@nus.edu.sg (Y.-K.L.); p.ross@ucc.ie (R.P.R.); catherine.stanton@teagasc.ie (C.S.); 4 Department of Microbiology & Immunology, National University of Singapore, Singapore 117545, Singapore; wei.t.khine@utu.fi; 5 Department of Food and Agricultural Product Technology, Universitas Gadjah Mada, Yogyakarta 55281, Indonesia; endangsrahayu@ugm.ac.id; 6 APC Microbiome Ireland, University College Cork, T12 K8AF Cork, Ireland; 7 Teagasc Food Research Centre, Moorepark, Fermoy, P61 C996 Cork, Ireland; 8 National Engineering Research Center for Functional Food, Jiangnan University, Wuxi 214122, China; 9 Wuxi Translational Medicine Research Center and Jiangsu Translational Medicine Research Institute Wuxi Branch, Wuxi 214122, China

**Keywords:** human breast milk, maternal feces, infant feces, *Bifidobacterium* community, *Lactobacillus* community

## Abstract

The infant gut microbiota plays a critical role in early life growth and derives mainly from maternal gut and breast milk. This study aimed to analyze the differences in the gut microbiota, namely *Bifidobacterium* and *Lactobacillus* communities at species level among breast milk as well as maternal and infant feces at different time points after delivery. Fifty-one mother–infant pairs from Indonesia were recruited, and the breast milk and maternal and infant feces were collected and analyzed by high throughput sequencing (16S rRNA, *Bifidobacterium* groEL and *Lactobacillus* groEL genes). PCoA results showed bacterial composition was different among breast milk and maternal and infant feces within the first two years. The abundance of *Bifidobacterium* and *Bacteroides* were significantly higher in infant feces compared to their maternal feces from birth to two years of age, and maternal breast milk within six months after birth (*p <* 0.05), whereas the abundance of *Blautia*, *Prevotella*, and *Faecalibacterium* was higher in maternal feces compared to that in breast milk within six months and infant feces within one year after birth, respectively (*p <* 0.05). The relative abundances of *Bacteroides* and *Lactobacillus* was higher and lower in infant feces compared to that in maternal feces only between one and two years of age, respectively (*p <* 0.05). For *Bifidobacterium* community at species level, *B. adolescentis, B. ruminantium, B. longum subsp. infantis, B. bifidum,* and *B. pseudolongum* were identified in all samples. However, the profile of *Bifidobacterium* was different between maternal and infant feces at different ages. The relative abundances of *B. adolescentis* and *B. ruminantium* were higher in maternal feces compared to those in infant feces from birth to one year of age (*p <* 0.05), while the relative abundances of *B. longum* subsp. *infantis* and *B. bifidum* were higher in infant feces compared to those in maternal feces beyond three months, and the relative abundance of *B. pseudolongum* was only higher in infant feces between three and six months (*p <* 0.05). For *Lactobacillus* community, *L. paragasseri* showed higher relative abundance in infant feces when the infant was younger than one year of age (*p <* 0.05). This study showed bacterial composition at the genus level and *Bifidobacterium* and *Lactobacillus* communities at the species level were stage specific in maternal breast milk as well as and maternal and infant feces.

## 1. Introduction

The gut microbiota in developing infants plays an important role in improving, regulating, and maintaining early life health [1,2]. Therefore, the first 1000 days represent a crucial window for healthy early life growth and development [3]. From birth to six months, the infant gut microbiota is mainly defined by mode of delivery (cesarean or vaginal delivery), feeding method, lactation stage, and maternal microbiota. With the introduction of solid food from six months onward, the gut microbiota deviates from that during the first six months [4]. Various bacterial compositions have been seen at different ages. However, the core bacterium in the infant gut remains *Bifidobacterium* before weaning, and with the introduction of solid food, the structure and dynamics of infant/child bacteria become more adult-like. In addition to *Bifidobacterium*, *Cutibacterium* which has the ability to metabolize lactic acid and produce propionic acid, is also an early colonizer in the infant gut. The relative abundance of *Cutibacterium* has been shown to decrease with age [5], while *Clostridia*, *Dialister*, and *Akkermansia* cannot be detected at birth but occur in the infant gut from 12 months onward [6].

The source of the infant gut microbiota varies, mainly arising from maternal gut, human breast milk (HBM), vagina, mammary gland, and the environment [7], among which maternal gut and HBM were reported to account for 22.1% and 18.5% of infant gut microbiota, respectively [8,9]. The gut microbiota of vaginally delivered and breast-fed infants were found to be more similar to that of maternal intestines and HBM, while the gut microbiota of infants delivered by caesarean section were more similar to that of skin of maternal mammary gland [7]. However, some recent studies have indicated that infant gut microbiota could be different from maternal gut and HBM although they were vaginally delivered and exclusively breast-fed [10,11]. For instance, the infant gut was colonized largely by *Bifidobacterium* and *Bacteroides* within the first six months, while the most common bacteria in HBM were *Staphylococcus* and *Streptococcus* and with a low level of *Bifidobacterium* [3], while the maternal intestine was dominated by *Prevotella* [12]. Additionally, the richness and diversity of bacteria in the maternal gut have been reported as seen significantly higher compared to that in HBM and the infant gut, while the maternal gut had less *Bacteroides* and a higher relative abundance of *Clostridia* class compared to the infant gut [13].

A study on the Indonesian infant fecal microbiome from birth until weaning showed a different microbiome from their mothers [12]. The purpose of the present study was to track HBM and gut bacterial communities of mothers and their infants at species level at different time points after delivery. In the present study, 51 mother–infant pairs, delivered vaginally, full term, and exclusively breastfed, were recruited and sampled from birth to two years of age in Indonesia. The 133 samples (31 HBM samples, 51 maternal feces, and 51 child feces) were analyzed for microbiota at genus level based on 16S rRNA sequencing, while *Bifidobacterium* and *Lactobacillus* communities and abundance at species level were enumerated by *Bifidobacterium* and *Lactobacillus* groEL sequencing, respectively.

## 2. Materials and Methods

### 2.1. Recruitment of Volunteers

A total of 51 mother–infant pairs in Indonesia were recruited in this study. The Universitas Gadjah Mada’s review board approved this study (Ref # KE/FK/335/EC, 8 April 2014). Before participation, informed consent was obtained from all individuals and all experiments were performed according to approved guidelines and regulations.

The recruited mothers had no history of serious illness. All the infants were vaginally delivered and exclusively breast fed to six months old age. Both mothers and children did not receive drugs, antibiotics, nor probiotics throughout the study. The maternal–infant pairs were divided into five groups according to the age of child, namely <one month (*n* = 10 pairs), 1–three months (*n* = 11 pairs), 3–six months (*n* = 10 pairs), 6–12 months (*n* = 10 pairs), and 12–24 months (*n* = 10 pairs).

### 2.2. Collection of Samples and Sample Processing

#### 2.2.1. HBM Samples

HBM samples were collected before six months after delivery. The skin of maternal breasts was scrubbed with alcohol and the first 1–2 mL breast milk were discarded to minimize potential contamination. A total of 10 mL breast milk was collected in a 50 mL sterile tube and stored frozen at −80 °C. HBM samples were defrosted at room temperature and centrifuged at 4 °C (10,000× *g*/min, 10 min) to remove fat and whey. Sediments were collected for DNA extraction.

#### 2.2.2. Fecal Samples

Fecal samples of mothers and children from 1 to 24 months of age after birth were collected. Hence, we collected 5 g of feces and suspended in a 15 mL tube. Then, 1 g fecal samples were transferred to 15 mL tubes and 10 mL phosphate buffered solution was added before centrifugation at 4 °C (1000× *g*/min, 5 min) to remove large fragments. Then, 5 mL supernatants were moved to a new tube and centrifuged at 4 °C (10,000× *g*/min, 10 min) to collect sediments for DNA extraction.

### 2.3. DNA Extraction and High throughput Sequencing

FastDNA Spin Kit for Feces (MP Biomedicals, LLC, Irvine, CA, USA) was used for DNA extraction from HBM and fecal samples. Bacterial sediment was put into a Lysing Matrix E tube. Then, 825 μL sodium phosphate buffer and 275 μL pre-lysis solution dissolving solution were added and vortexed for 10–15 s, followed by centrifugation at 14,000× *g* for 5 min, and the supernatant was discarded. Subsequently, 978 μL sodium phosphate buffer and 122 μL MT buffer were added and to the mix, shaken, and broken at 70 HZ on the high-throughput tissue grinder for 30 s (3~5 times). Then, the Lysing Matrix E tube was centrifuged at 14,000× *g* for 10 min. Finally, bacterial DNA was in supernatant and purified utilizing the FastDNA Spin Kit for Feces.

### 2.4. 16S rRNA Sequence

The V3-V4 regions of the 16S rRNA gene was PCR-amplified using primers 341F (5′-CCTAYGGGRBGCASCAG-3′) and 806R (5′-GGACTACNNGGGTATCTAAT-3′) [14]. The condition and system of PCR were referenced as previously described [15]. The PCR product was loaded to agarose gel electrophoresis. Then QIAquick Gel Extraction Kit (QIAGEN, Hilden, Germany) was used for the recovery of electrophoretic gel products. After the extracted products were determined by the Qubit 3.0. fluorometer, DNA libraries were loaded on an Illumina MiSeq sequencing system according to the manufacturer’s instructions.

### 2.5. GroEL Sequencing of Bifidobacterium and Lactobacillus

The step for groEL sequencing of *Bifidobacterium* and *Lactobacillus* was similar to that in 16S rRNA sequencing except with different primers and PCR conditions according to the previous description [16].

### 2.6. Statistical Analysis

The QIIME 2 data analysis software package was used to analyze the 16S rRNA data [17] as previously described [14]. The sequence of *Bifidobacterium* and *Lactobacillus* groEL genes was assigned by the *Bifidobacterium* groEL database and *Lactobacillus groEL* database from a previous study [16]. Sequences of 16S rRNA, *Bifidobacterium* groEL, and *Lactobacillus* groEL were used to calculate the alpha diversity. Beta diversity was assessed by principal coordinate analysis (PCoA). The distance among groups was estimated from the Bray–Curtis index, and PERMANOVA was used to calculate the difference [18]. The differences of alpha diversity and the relative abundance of bacteria between two groups were quantified by the paired T test and R package.

## 3. Results

### 3.1. Bacterial Diversity of HBM, Maternal Feces and Child Feces

**Figure 1:**
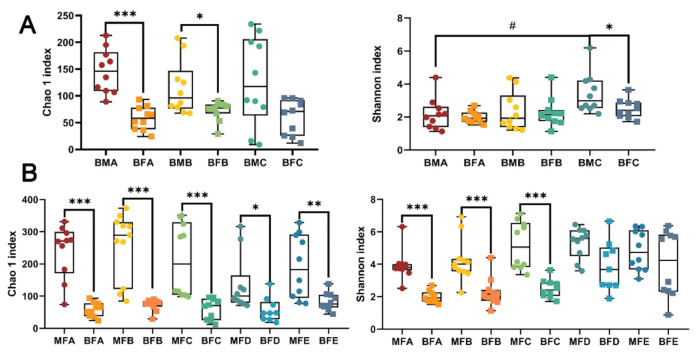
Alpha-diversity illustrating richness and diversity in microbiota of human breast milk and infant feces (**A**), maternal and infant feces (**B**). *, *p <* 0.05; ** *p <* 0.01, *** *p <* 0.001; ^#^ *p* < 0.05. BMA: breast milk (corresponding to infant younger than one month of age), BMB: breast milk (corresponding to infant between 1 and three months of age), BMC: breast milk (corresponding to infant between three and six months of age), MFA: maternal feces (corresponding to infant younger than one month of age), MFB: maternal feces (corresponding to infant between 1 and three months of age), MFC: maternal feces (corresponding to infant between three and six months of age), MFD: maternal feces (corresponding to infant between six and 12 months of age), MFE: maternal feces (corresponding to infant older than 12 months of age), BFA: infant feces younger than one month of age, BFB: infant feces between 1 and three months of age, BFC: infant feces between three and six months of age, BFD: infant feces between six and 12 months of age, BFE: infant feces older than 12 months of age.

Chao1 and Shannon indices were used to assess the richness and diversity of the bacterial community, respectively. BMA group (infant younger than one month of age) showed a significantly higher Chao 1 index compared to that of BFA group (*p <* 0.001, Figure 1A), and BMB group (infant between one and three months of age) showed a significantly higher Chao 1 index compared to that of BFB group (*p <* 0.05, Figure 1A), while infants in BMC group (between three and six months of age) showed higher diversity compared to BFC group based on Shannon index (*p <* 0.05, Figure 1A). In addition, alpha diversity in bacterial communities between the maternal–child feces, group MFA, MFB, and MFC showed higher diversity and richness compared to those in BFA, BFB, and BFC, respectively (*p <* 0.001, Figure 1B). Additionally, a higher richness was found in MFD and MFE groups compared to BFD (infant between six and 12 months of age, *p <* 0.05) and BFE (infant older than 12 months of age, *p <* 0.01, Figure 1B), while no significant difference in diversity was found between them.

**Figure 2:**
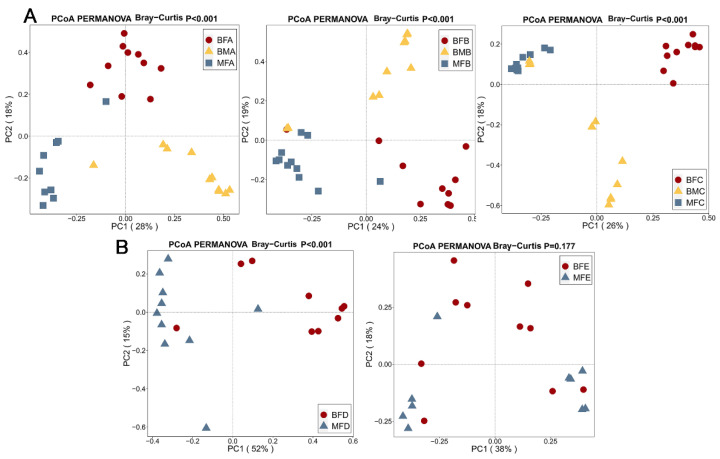
Beta-diversity illustrating composition in microbiota of of human breast milk, maternal and infant feces. (**A**) samples collected before six months of age. (**B**) samples collected between six and 24 months of age. PERMANOVA was used to calculate the difference among samples based on Bray–Curtis distance. *p* < 0.05 stands for significantly different microbiota composition was found among samples. BMA: breast milk (corresponding to infant younger than one month of age), BMB: breast milk (corresponding to infant between 1 and three months of age), BMC: breast milk (corresponding to infant between three and six months of age), MFA: maternal feces (corresponding to infant younger than one month of age), MFB: maternal feces (corresponding to infant between 1 and three months of age), MFC: maternal feces (corresponding to infant between three and six months of age), MFD: maternal feces (corresponding to infant between six and 12 months of age), MFE: maternal feces (corresponding to infant older than 12 months of age), BFA: infant feces younger than one month of age, BFB: infant feces between 1 and three months of age, BFC: infant feces between three and six months of age, BFD: infant feces between six and 12 months of age, BFE: infant feces older than 12 months of age.

PCoA was carried out to further evaluate the dissimilarity of bacterial composition among HBM, maternal, and child feces based on the Bray–Curtis metric. Significant differences were found in the bacterial composition of HBM, maternal, and children fecal samples 1–six months after birth (*p <* 0.001) based on Bray–Curtis distance (Figure 2A). In addition, similar differences in bacterial composition were found between maternal and child feces 6–12 months after birth (*p <* 0.001, Figure 2B). However, the differences disappeared when the children were older than 12 months (*p =* 0.177, Figure 2B).

### 3.2. Bacterial Composition and Difference among HBM, Maternal Feces and Child Feces

**Figure 3:**
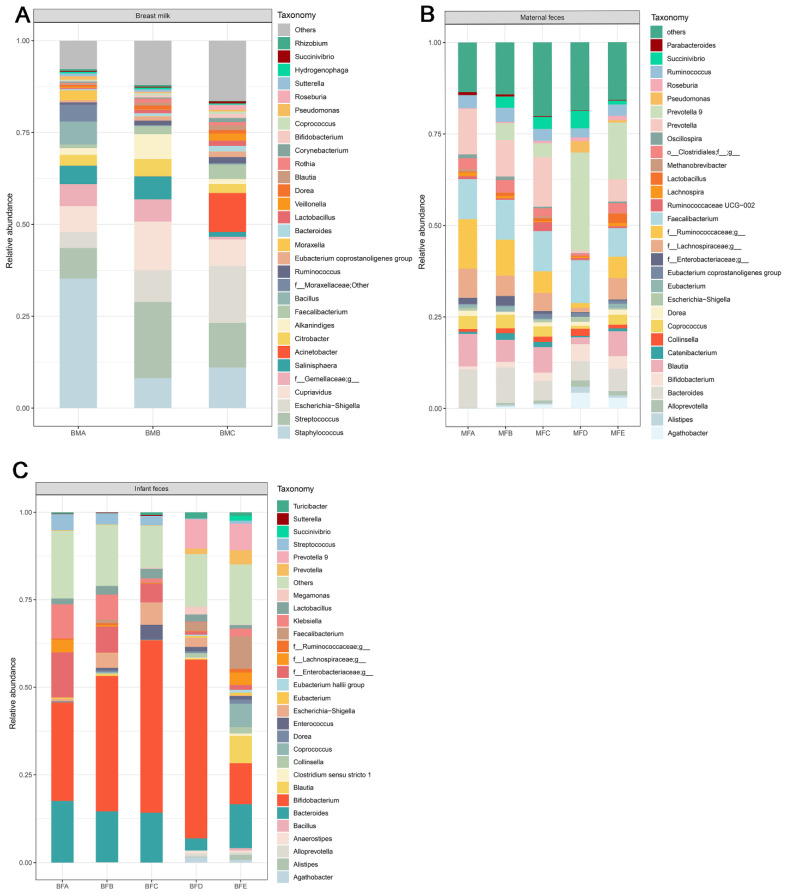
Top 30 genera in human breast milk, maternal and infant feces. (**A**) human breast milk; (**B**) maternal feces; (**C**) child’s feces. BMA: breast milk (corresponding to infant younger than one month of age), BMB: breast milk (corresponding to infant between 1 and three months of age), BMC: breast milk (corresponding to infant between three and six months of age), MFA: maternal feces (corresponding to infant younger than one month of age), MFB: maternal feces (corresponding to infant between 1 and three months of age), MFC: maternal feces (corresponding to infant between three and six months of age), MFD: maternal feces (corresponding to infant between six and 12 months of age), MFE: maternal feces (corresponding to infant older than 12 months of age), BFA: infant feces younger than one month of age, BFB: infant feces between 1 and three months of age, BFC: infant feces between three and six months of age, BFD: infant feces between six and 12 months of age, BFE: infant feces older than 12 months of age.

OTU analysis revealed the bacterial composition in the HBM and maternal and children fecal samples. The top 30 bacteria in each stage of HBM and maternal and child fecal samples were selected. The other genera were classified as ‘other’. *Staphylococcus* (35.2%), *Streptococcus* (20.7%), and *Escherichia-Shigella* (15.5%) were predominant in HBM samples at different stages throughout lactation, namely BMA, BMB, and BMC (Figure 3A). *Bifidobacterium* showed a low relative abundance among the three stages of HBM samples (0.15%, 0.5%, 1.1%) (Figure 3A). Low relative abundance was also found in *Lactobacillus* at the respective stages (0.3%, 0.9%, 1.4%). The low abundances may result in a low level of amplification for *Bifidobacterium* and *Lactobacillus* and difficulty in identification at the species level of HBM samples (Figure S1A).

Among the top 30 genera, Ruminococcaceae was predominant in MFA (13.5%), *Faecalibacterium* was predominant in MFB (10.9%) and MFC (11.0%), and *Prevotella* was predominant in MFD (26.8%) and MFE (15.5%). *Bifidobacterium* and *Lactobacillus* were also among the top 30 genera. *Bifidobacterium* showed the lowest relative abundance in MFA (0.8%) and higher in MFB (1.5%), MFC (2.3%), MFD (4.7%), and MFE (3.4%). The relative abundance of *Lactobacillus* was lower than 1% in MFA (0.3%), MFB (0.9%), MFC (0.8%), and MFD (0.2%), while the relative abundance of *Lactobacillus* in MFE group was 2.6% (Figure 3B). 

The predominant genus found in child feces was *Bifidobacterium* regardless of age in the current study. The relative abundance of *Bifidobacterium* in the different age stages were 28.0%, 38.6%, 49.2%,59.0%, and 11.7%, respectively. However, *Lactobacillus* in child feces showed low relative abundance throughout all the groups, which was 1.7%, 2.5%, 2.8%, 2.1%, and 1.0%, respectively.

### 3.3. Bacterial Difference among HBM, Maternal and Child Feces

**Figure 4:**
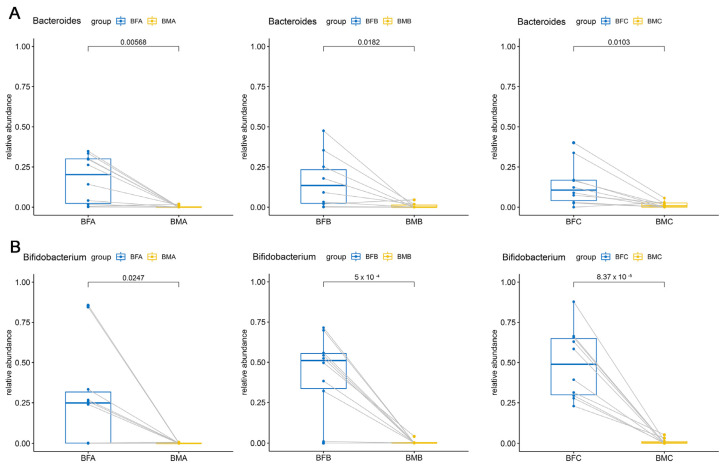
Different genera in human breast milk and infant feces. (**A**), significant difference of *Bacteroides* in HBM and infant feces. (**B**) significant difference of *Bifidobacterium* in HBM and infant feces. The two points connected by a line are the same mother-infant pair. BMA: breast milk (corresponding to infant younger than one month of age), BMB: breast milk (corresponding to infant between one and three months of age), BMC: breast milk (corresponding to infant between three and six months of age), BFA: infant feces younger than one month of age, BFB: infant feces between one and three months of age, BFC: infant feces between three and six months of age, BFD: infant feces between six and 12 months of age, BFE: infant feces older than 12 months of age.

Orthogonal partial least squares discrimination analysis (OPLS-DA) was performed to investigate the inherent differences among HBM, maternal and children fecal samples. According to the VIP and *p* values of the assigned microbiota (VIP > 1, *p <* 0.05), from one to six months, higher relative abundances of *Bacteroides* and *Bifidobacterium* were found in infant feces compared to HBM (*Bacteroides*: BFA vs. BMA, *p <* 0.05; BFB vs. BMB, *p <* 0.05; BFC vs. BMC, *p <* 0.05; *Bifidobacterium*: BFA vs. BMA, *p <* 0.05; BFB vs. BMB, *p <* 0.001; BFC vs. BMC, *p <* 0.001; Figure 4). 

**Figure 5:**
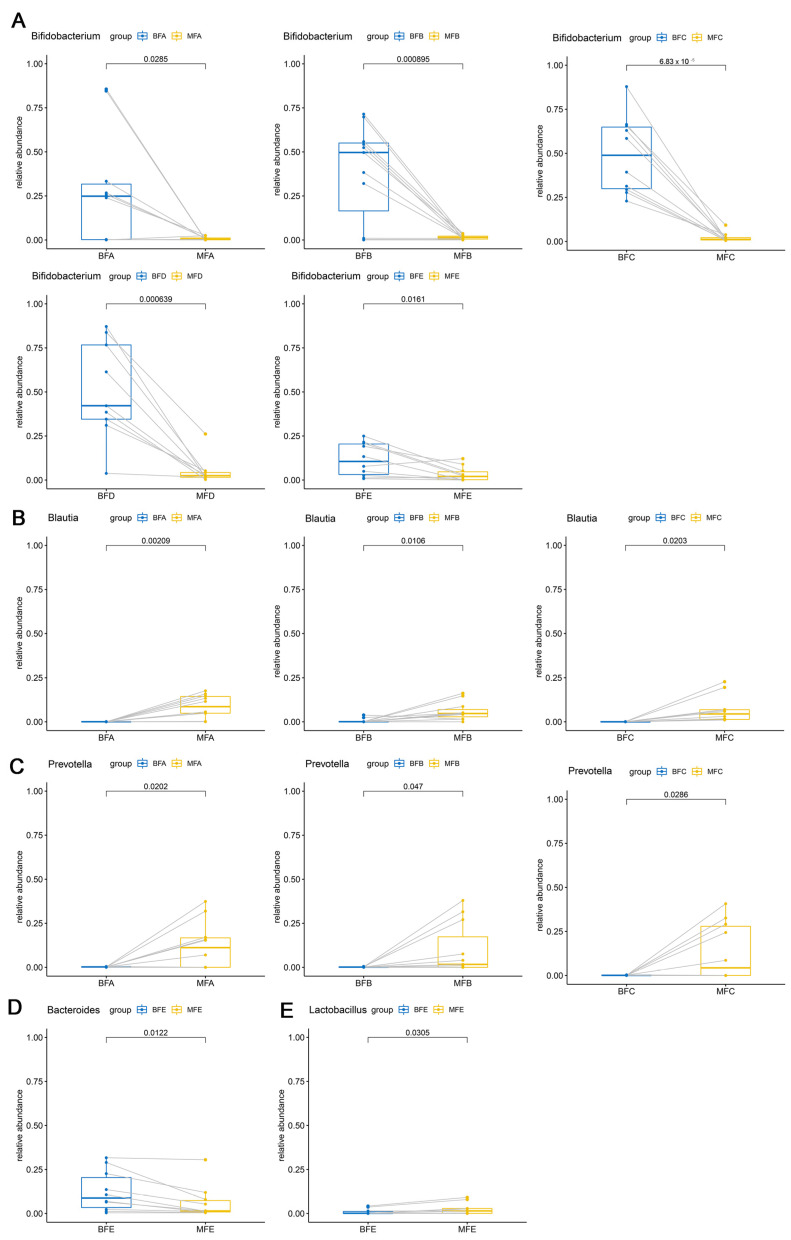
Different genera in maternal and infant feces. (**A**–**E**) stand for significant difference of *Bifidobacterium*, *Blautia*, *Prevotella*, *Bacteroides*, and *Lactobacillus* in maternal and infant feces, respectively. The two points connected by a line are the same mother-infant pair. MFA: maternal feces (corresponding to infant younger than one month of age), MFB: maternal feces (corresponding to infant between one and three months of age), MFC: maternal feces (corresponding to infant between three and six months of age), MFD: maternal feces (corresponding to infant between six and 12 months of age), MFE: maternal feces (corresponding to infant older than 12 months of age), BFA: infant feces younger than one month of age, BFB: infant feces between one and three months of age, BFC: infant feces between three and six months of age, BFD: infant feces between six and 12 months of age, BFE: infant feces older than 12 months of age.

**Figure 6:**
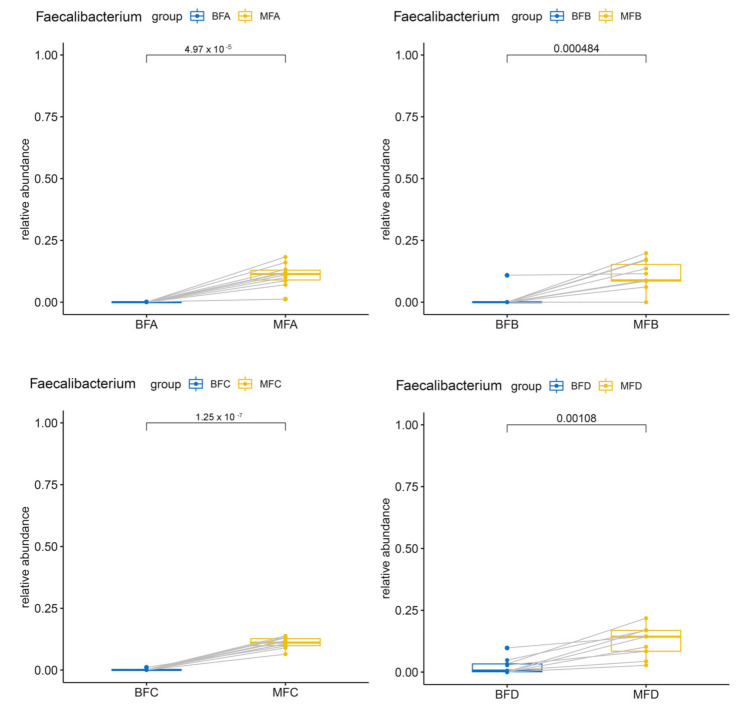
Different *Faecalibacterium* in maternal and infant feces. The two points connected by a line are the same mother-infant pair. MFA: maternal feces (corresponding to infant younger than one month of age), MFB: maternal feces (corresponding to infant between one and three months of age), MFC: maternal feces (corresponding to infant between three and six months of age), MFD: maternal feces (corresponding to infant between six and 12 months of age), MFE: maternal feces (corresponding to infant older than 12 months of age), BFA: infant feces younger than one month of age, BFB: infant feces between one and three months of age, BFC: infant feces between three and six months of age, BFD: infant feces between six and 12 months of age, BFE: infant feces older than 12 months of age.

The *Bifidobacterium* community also showed significant difference between maternal and child feces among the five stages (BFA vs. MFA, *p <* 0.05; BFB vs. MFB, *p <* 0.001; BFC vs. MFC, *p <* 0.001; BFD vs. MFD, *p <* 0.001; BFE vs. MFE, *p <* 0.05; Figure 5A). For *Blautia* and *Prevotella*, significant difference between maternal and child feces were only found from the first month to sixth month after birth (Blautia: BFA vs. MFA, *p <* 0.01; BFB vs. MFB, *p <* 0.05; BFC vs. MFC, *p <* 0.05; Figure 5B; *Prevotella*: BFA vs. MFA, *p <* 0.05; BFB vs. MFB, *p <* 0.05; BFC vs. MFC, *p <* 0.05; Figure 5C). However, from the 12th month to the 24th month, a higher relative abundance of *Bacteroides* and a lower relative abundance of *Lactobacillus* were found in BFE (*p <* 0.05, Figure 5D; *p <* 0.05, Figure 5E). Additionally, the relative abundance of *Faecalibacterium* was higher in maternal feces than that in child feces, except for children over 12 months of age (BFA vs. MFA, *p <* 0.001; BFB vs. MFB, *p <* 0.001; BFC vs. MFC, *p <* 0.001; BFD vs. MFD, *p <* 0.01; Figure 6).

### 3.4. Diversity and Composition of Bifidobacterium Community in Maternal and Child Feces

Fifty-one maternal feces and their child feces in pairs were used to amplify *Bifidobacterium* composition at species level, among which only 47 maternal and 42 children fecal samples were amplified successfully (BFA: *n* = 7; MFA: *n* = 9; BFB: *n* = 8, MFB: *n* = 9; BFC *n* = 9, MFC: *n* = 10; BFD: *n* = 9, MFD: *n* = 10; BFE: *n* = 9, MFE: *n* = 9). All the HBM samples failed to be amplified.

Based on *Bifidobacterium* groEL sequence, Chao 1 and Shannon index were used to assess the richness and diversity of *Bifidobacterium* between maternal and children fecal samples. No significant difference was observed between maternal feces and child feces among all the five stages (Figure S1A). 

**Figure 7:**
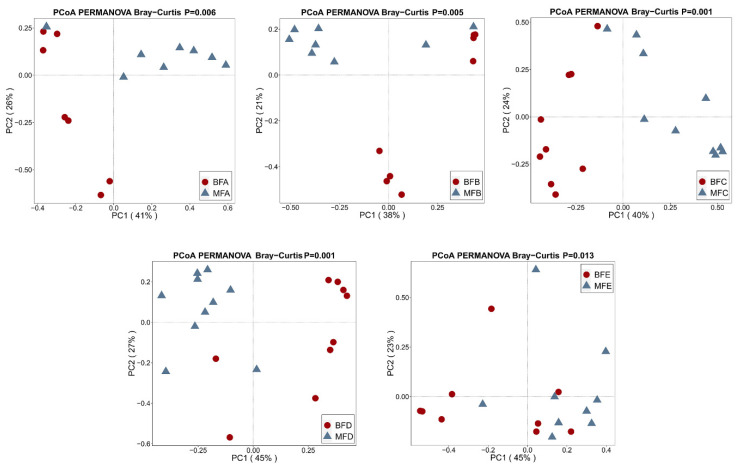
Beta-diversity illustrating of *Bifidobacterium* community between maternal and infant feces. PERMANOVA was used to calculate the difference among samples based on Bray–Curtis distance. *p* < 0.05 stands for significantly different microbiota composition was found among samples. MFA: maternal feces (corresponding to infant younger than one month of age), MFB: maternal feces (corresponding to infant between one and three months of age), MFC: maternal feces (corresponding to infant between three and six months of age), MFD: maternal feces (corresponding to infant between six and 12 months of age), MFE: maternal feces (corresponding to infant older than 12 months of age), BFA: infant feces younger than one month of age, BFB: infant feces between one and three months of age, BFC: infant feces between three and six months of age, BFD: infant feces between six and 12 months of age, BFE: infant feces older than 12 months of age.

PCoA was utilized to present the dissimilarity of *Bifidobacterium* composition in maternal feces and child feces. From birth to 12 months old, *Bifidobacterium* composition in child feces was significantly different from that in maternal feces (BFA vs. MFA, *p =* 0.006; BFB vs. MFB, *p =* 0.005; BFC vs. MFC, *p =* 0.001; BFD vs. MFD, *p =* 0.001; BFE vs. MFE, *p =* 0.013; Figure 7). 

The database of *Bifidobacterium* groEL was used to assign sequence and 18 *Bifidobacterium* species were detected, and the relative abundance of *Bifidobacterium* species lower than 0.001% was classified as *Bifidobacterium* ‘other’ [16]. *B. longum* subsp. *longum* was predominant in child feces (BFA: 43.6%, BFB: 44.5%, BFC: 33.4%, BFD: 43.0%, BFE: 32.9%), followed by *B. breve*, *B. bifidum*, *B. pseudocatenulatum*, and *B. longum* subsp. *infantis*. The relative abundance of other *Bifidobacterium* species was less than 5% on average. However, the predominant *Bifidobacterium* species in maternal feces was *B. adolescentis* (MFA: 40.5%, MFB: 33.9%, MFC: 26.0%, MFD: 39.4%, MFE: 48.2%) followed by *B. longum* subsp. *longum*, *B. pseudocatenulatum*, *B. ruminantium*, and the other species, which was less than 5% on average (Figure S1B).

### 3.5. Bifidobacterium Difference between Maternal and Child Feces at Species Level

**Figure 8:**
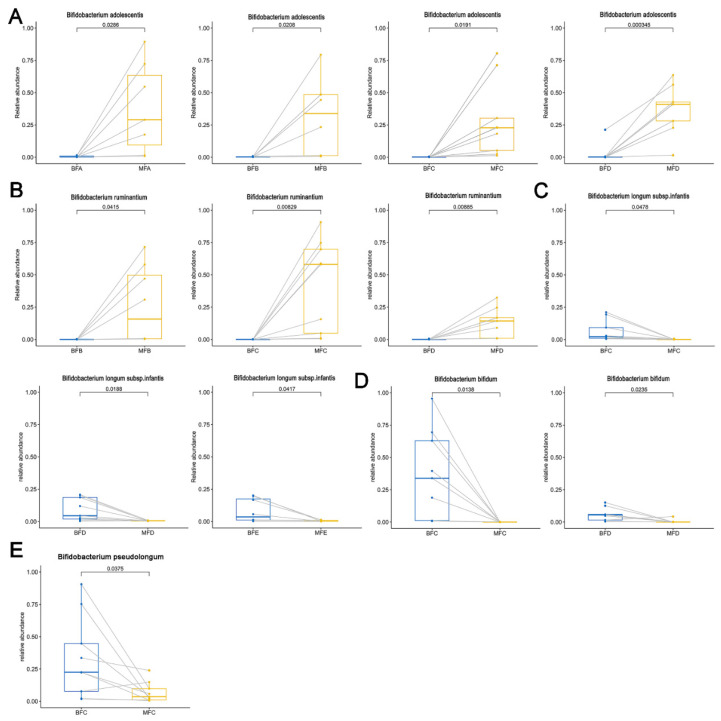
Differences of *Bifidobacterium* at species level in maternal and infant feces. (**A**–**E**) stand for significant difference of *Bifidobacterium adolescentis*, *Bifidobacterium ruminantium*, *Bifidobacterium longum* subsp. *infantis*, *Bifidobacterium bifidum*, and *Bifidobacterium pseudolongum* in maternal and infant feces, respectively. The two points connected by a line are the same mother-infant pair. MFA: maternal feces (corresponding to infant younger than one month of age), MFB: maternal feces (corresponding to infant between 1 and three months of age), MFC: maternal feces (corresponding to infant between three and six months of age), MFD: maternal feces (corresponding to infant between six and 12 months of age), MFE: maternal feces (corresponding to infant older than 12 months of age), BFA: infant feces younger than one month of age, BFB: infant feces between 1 and three months of age, BFC: infant feces between three and six months of age, BFD: infant feces between six and 12 months of age, BFE: infant feces older than 12 months of age.

Samples that were successfully amplified in both maternal and child feces were used for paired comparison. Higher relative abundance of *B. adolescentis* was detected in maternal feces than that in in child feces from birth to 12 months of age (BFA vs. MFA, *p <* 0.05; BFB vs. MFB, *p <* 0.05; BFC vs. MFC, *p <* 0.05; BFD vs. MFD, *p <* 0.001; Figure 8A) and this difference disappeared when the children were older than 12 months. However, there was no difference in the relative abundance of *B. ruminantium* between the feces of mothers and children from birth to one month of age and 6–12 months of age, but the relative abundance of *B. ruminantium* in maternal feces was significantly higher than that in child feces from one to 12 months of age (BFB vs. MFB, *p <* 0.05; BFC vs. MFC, *p <* 0.01; BFD vs. MFD, *p <* 0.01; Figure 8B). In contrast to *B. ruminantium*, *B. longum* subsp. *longum* showed a higher abundance in child feces than that in maternal feces from one to 12 months of age (BFB vs. MFB, *p <* 0.05; BFC vs. MFC, *p <* 0.01; BFD vs. MFD, *p <* 0.05; Figure 8C). In addition, *B. bifidum* was found at a higher relative abundance in child feces than that in maternal feces from three to 12 months of age (BFC vs. MFC, *p <* 0.05; BFD vs. MFD, *p <* 0.05; Figure 8D).

### 3.6. Diversity and Composition of Lactobacillus Community in Maternal and Child Feces

By *Lactobacillus* groEL gene sequencing, fifty-one maternal/children fecal pairs were amplified for *Lactobacillus* profile analysis, among which only 27 maternal and 34 children samples were amplified successfully (BFA: *n* = 3; MFA: *n* = 6; BFB: *n* = 5, MFB: *n* = 8; BFC *n* = 6, MFC: *n* = 9; BFD: *n* = 7, MFD: *n* = 4; BFE: *n* = 6, MFE: *n* = 7), which might be resulted from low relative abundance of *Lactobacillus*. For HBM samples, none sample was amplified successfully for *Lactobacillus* identification.

**Figure 9:**
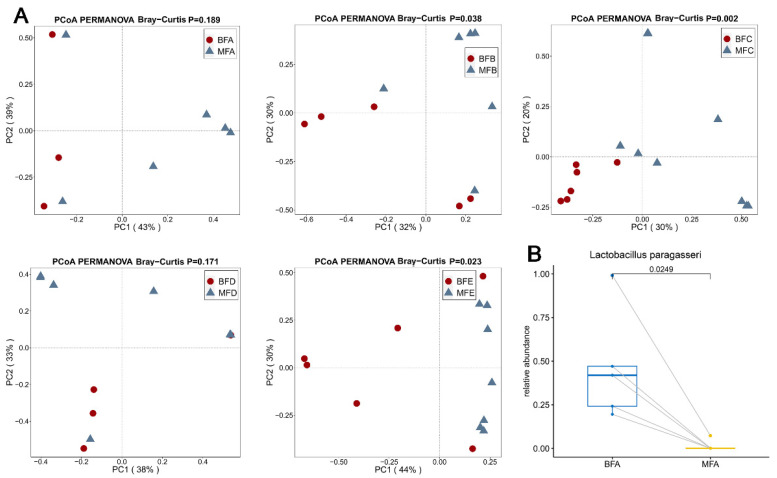
Beta-diversity illustrating of *Lactobacillus* community in maternal and children feces (**A**). Difference in *Lactobacillus* community in maternal and infant feces (**B**). PERMANOVA was used to calculate the difference among samples based on Bray-Curtis distance. Difference of *Lactobacillus* at species level between maternal and infant feces (**B**). MFA: maternal feces (corresponding to infant younger than one month of age), MFB: maternal feces (corresponding to infant between 1 and three months of age), MFC: maternal feces (corresponding to infant between three and six months of age), MFD: maternal feces (corresponding to infant between six and 12 months of age), MFE: maternal feces (corresponding to infant older than 12 months of age), BFA: infant feces younger than one month of age, BFB: infant feces between 1 and three months of age, BFC: infant feces between three and six months of age, BFD: infant feces between six and 12 months of age, BFE: infant feces older than 12 months of age.

Using the same method for diversity analysis as performed previously, MFB and MFE groups showed significantly higher diversity compared to BFB and BFE based on Shannon index, respectively (*p* < 0.05, Figure S2). No significant difference in richness was found between maternal and children fecal samples based on Chao 1 index. PCoA results showed the *Lactobacillus* composition of child feces was significantly different to maternal feces from one to six months, and from 12 to 24 months (*p =* 0.038, *p =* 0.002, *p =* 0.023, respectively, Figure 9A). However, when children were younger than one month of age, and within 6–12 months of age, the *Lactobacillus* composition in child feces was similar to that in maternal feces (*p =* 0.189, *p =* 0.171, respectively, Figure 9A).

The database of *Lactobacillus* groEL genes was used to assign the sequence, and 54 *Lactobacillus* species were identified [16]. The relative abundance of *Lactobacillus* species less than 0.001% was classified as *Lactobacillus* other. Only 16 *Lactobacillus* species were presented (Figure S2B). The feces of children showed different dominant *Lactobacillus* species at different ages. *L. paragasseri* was predominant in child feces of BFA (44.2%), BFC (38.6%), and BFE (28.5%, Figure S2B), whereas in BFB and BFD, the predominant *Lactobacilli* were *L. paracasei* (34.5%) and *L. mucosae* (28.6%), respectively. For *Lactobacillus* composition of maternal feces, the early three stages were dominated by *L. mucosae*, namely MFA (39.8%), MFB (43.8%), and MFD (40.2%) and in the latter two stages, *L. ruminis* was dominant (MFC: 37.6%); MFE: 45.1%; Figure S2B).

### 3.7. Lactobacillus Difference between Maternal and Child Feces at Species Level

All the *Lactobacillus* species in maternal and child feces were compared based on the paired T test. Only *L. paragasseri* showed a significantly higher relative abundance in BFA compared to that in MFA (*p <* 0.05, Figure 9B).

## 4. Discussion

In this study, 51 mother–infant pairs in Indonesia were recruited and 133 samples (31 HBM samples, 51 maternal feces and 51 child feces) were used to assess the microbiota composition at genus level and *Bifidobacterium* and *Lactobacillus* communities at species level based on 16S rRNA sequencing and *Bifidobacterium* and *Lactobacillus* groEL sequencing. *Bifidobacterium* and *Lactobacillus* composition at species level proved to be age-dependent in the three types of samples. Among the *Bifidobacterium* community, *B. adolescentis*, *B. ruminantium*, *B. longum* subsp. *infantis*, and *B. bifidum* were all detected but showed different relative abundance in maternal and child feces. For the *Lactobacillus* community, *L. paragasseri* showed significantly higher relative abundance in BFA compared to that in MFA.

*Staphylococcus* and *Streptococcus* have been regarded as the dominant bacteria in HBM [19]. In our study, *Staphylococcus* was detected in three age stages of HBM, but only dominant in BMA group, whose age was younger than one month. When the children were older than one month, the dominant genera were *Streptococcus* and *Escherichia-Shigella*, in which *Escherichia-Shigella* was commonly found in human feces [20]. This may indicate that bacteria in the maternal gut could enter into the mammary gland via the entero-mammary pathway [21]. However, we could not rule out the possibility of fecal–skin contamination. Earlier research suggested the core microbiota of HBM was composed of *Ralstonia*, *Flavobacterium*, *Propionibacterium*, *Burkholderia*, *Rothia*, *Bifidobacterium*, *Corynebacterium*, *Blautia*, and *Brevundimonas* [22] within four weeks after delivery, while *Bifidobacterium*, *Flavobacterium*, *Lactobacillus*, *Stenotrophomonas*, *Brevundimonas*, *Chryseobacterium*, and *Enterobacter* were detected within three months after delivery [23]. Maternal dietary and health status, in addition to technical deviation, may contribute to the discrimination of core bacteria in HBM [24,25]. Additionally, *Bifidobacterium* and *Lactobacillus*, often used as probiotics, can be detected in HBM in our study with low relative abundance which is consistent with previous report [12]. The low relative abundance in our study may have caused the failure to amplify *Bifidobacterium* and *Lactobacillus* at species level.

Different from microbiota in HBM, maternal fecal microbiota was dominated by Ruminococcaceae, *Faecalibacterium*, and *Prevotella* 9, which also showed age differentiation. The dominant microbiota of maternal feces changed before and after weaning. Ruminococcaceae and *Faecalibacterium* were predominant when the age of children was less than 1–six months of age, whereas after weaning, the dominant bacterium was *Prevotella* 9. In the high carbohydrate diet of the Indonesians, *Prevotella* was proliferate due to carbohydrate fermentation [26,27]. It would be interesting to verify if the changes in maternal diet or physiological stage before and after weaning may be the reason for different dominant bacteria in maternal feces.

Similar results were noted in dominant bacteria of child feces. There is no doubt that *Bifidobacterium* was the dominant bacteria in the infant gut [28], due to its ability to use human milk oligosaccharides, and this dominance can last until half a year after weaning and was consistent with a previous study where the hen infant gut was enriched with breast-milk-metabolizing bacteria (e.g., *Bifidobacterium*) [29]. It was then replaced by *Bacteroides* at a later age. In this case, microbiota in child feces was quite different from that in maternal feces and HBM both before and after weaning, as presented in the PCoA plot in this study when the child was younger than 12 months of age. This strongly suggests that the microbial composition in the gut of these infants was not directly inherited from and determined by the maternal fecal and breast milk microbiome. This is in agreement with a recent study [12]. However, microbiota of child feces was similar to maternal feces when children were older than 12 months of age. In addition, these results can also be reflected in the microbiota richness. The microbiota richness of maternal feces was always higher than that of infant feces, but this difference disappeared after weaning. These results indicated that, as the children consumed aa more similar diet to their mothers, their fecal microbiome became more similar to that of adults [12,30], although the dominant bacteria were still not the same as adults by the age of two years old. 

The results from animal experiments suggested that microbiota in HBM can be changed through adjusting the maternal diets [31]. However, we found that the different microbiota composition between HBM and child feces was not similar to the microbiota difference between maternal and child feces. The richness of microbiota in HBM was higher than that in child feces when children were younger than three months old, whereas the diversity of microbiota in HBM was higher than that of child feces when children were older than three months old. Meanwhile, *Bifidobacterium* showed lower relative abundance in HBM while higher relative abundance of *Bifidobacterium* was found in child feces. In this case, although microbiota in HBM, which was partly from maternal gut origin [32], and was a source of microbiota for the infants [33], the bacterial compositions in the HBM, maternal and child feces were significantly different. This may be explained by the differences in the ecological environment and intrinsic factors of infant gut in allowing selected bacteria to survive and strive. For HBM, microbiota may come from maternal gut [32], mammary gland [34] and infant oral sources [35] with the latter being relatively abundant in aerobic bacteria. For microbiota in child feces, microbiota from vagina was an additional source [8]. The majority of microbiota in vaginal area was *Lactobacillus* [36], whereas the relative abundance of *Lactobacillus* in child feces was lower than 5%. This suggested that microbiota types contributed from different maternal sources, while only selected microbiota could establish in the gut of infants and children. 

*Bifidobacterium* could be regarded as the core bacterium in the infant gut in this and earlier studies [37]. The function of *Bifidobacterium* and the reason for their prevalence in the gastrointestinal tract of children before weaning thus received much attention [12]. For instance, *B. animalis* subsp. *lactis* (Bb12) exhibited α-glucosidase inhibition activity and inhibited glucose absorption and transport [38]. *B. pseudocatenulatum* CECT 7765 could reduce the effect of chronic stress on the hypothalamic-pituitary-adrenal response of maternal separation in infancy, and this effect could persist into adulthood [39]. Thus, it could be a potential health risk for infants if they possess low relative abundance of *Bifidobacterium* as detected in Southeast Asians with *Prevotella* type gut microbiome [26,40]. Here, we found that the relative abundance of *Bifidobacterium* in Indonesian infants of the same age was lower than that in European infants [41,42]. Health-promoting functions of *Bifidobacterium* observed in European, North American, and East Asian Children [43,44] could be replaced by other symbiotic bacteria in Indonesian children, such as *Enterococcus* faecalis [45]. At the species level, *B. animalis* subsp. *lactis* cannot be detected in Indonesian infants in our study while it is abundant in Chinese infant feces [16,46]. *B. animalis* has been reported to prevent influenza infection [47].

In addition to the differences of *Bifidobacterium* community in the feces of children between Indonesia and other regions, the similarity and differences in *Bifidobacterium* species in the feces of children and their mothers were also observed. The Bifidobacterium community in Indonesian children was dominated by *B. longum* subsp. *longum*, *B. breve* and *B. bifidum,* while *B. longum* subsp. *longum*, *B. pseudocatenulatum*, *B. adolescentis* and *B. ruminantium* were dominant in maternal feces, which indicated that different *Bifidobacterium* species were favored in the gut of children and mothers. This age-dependent change of *Bifidobacterium* in Indonesian children was consistent with the differences in fecal *Bifidobacterium* species from infants and adults in China [16,37]. Additionally, the differences in *Bifidobacterium* composition at species level between maternal and child feces were continuous to the age of two in our study based on PCoA analysis. Meanwhile, the level of fecal *B. adolescentis* in Indonesian children from birth to the age of 12 months of age was lower than in maternal feces. This was in line with previous research suggesting that *B. adolescentis* was an adult-type species and also found in formula-milk fed infants [48]. *B. adolescentis* showed the ability to utilize starch and starchy carbohydrates through a genomic sequencing approach [49], which can explain its different abundance between Indonesian maternal and child feces, and the difference disappeared when the child was older than one year of age after weaning. *B. ruminantium* also showed a higher level in maternal feces compared with child feces at 1–12 months of age. Interestingly, *B. ruminantium* was mainly isolated from cattle [50], and a higher level of *B. ruminantium* was observed in Indonesian maternal feces, which may be explained by dietary habits and close vicinity to cattle during daily activities [17]. In contrast to the level of *B. ruminantium*, child feces showed a higher relative abundance of *B. longum* subsp. *infantis*, *B. bifidum,* and *B. pseudolongum* compared to that in maternal feces at different age stages. *B. longum* subsp. *infantis* and *B. bifidum* possessed the genes encoding enzymes for using HMOs specifically [51,52]. We found no difference in the abundance of these species between mothers and infants younger than three months of age, even the infants were exclusively breast fed. The differences in the abundance of *B. longum* subsp. *infantis* and *B. bifidum* between the mothers and infants only appeared after infants were older than three months. This could represent the lactation time for *B. longum* subsp. *infantis* and *B. bifidum*, which were the minority *Bifidobacterium* species in the mothers to flourish on HMOs, and became the predominant *Bifidobacterium* species in the gut of infants. 

*Lactobacillus* exhibited low relative abundance as reported in this and previous report [53]. Moreover, Indonesian maternal feces also had low levels of *Lactobacillus*. However, the dominant *Lactobacillus* species in maternal and infant feces were different. Maternal feces were dominated by *L. mucosae* and *L. ruminis,* while the infant feces were dominated by *L. paragasseri.* Additionally, *L. paragasseri* showed significantly higher abundance in infant younger than one month of age. The infants in this study were all vaginally delivered, and 70% of vaginal microbiota was *Lactobacillus,* [54]. Thus, *Lactobacillus* in the infant gut mainly came from maternal vagina [55] but not maternal gut due to the low abundance of *Lactobacillus* in the maternal gut. *Lactobacillus* is a beneficial microbe in the gut. For instance, *L. casei* could prevent childhood diarrhea through modulating gut microbiota [56] and *L. reuteri* could promote mucosal immune development [57]. The roles of these low abundant *Lactobacillus* in Indonesian infants remain unclear and need further investigation.

## 5. Conclusions

In conclusion, 51 mother–infant pairs in Indonesia were recruited and HBM and maternal and infant feces were collected to analyze the microbiota composition and *Bifidobacterium* and *Lactobacillus* community at the species level based on 16S rRNA sequencing and *Bifidobacterium* and *Lactobacillus* groEL sequencing. Bacterial composition at genus level and *Bifidobacterium* and *Lactobacillus* composition at species level showed age-dependent differences in the three types of samples and the relative abundance of *Bacteroides*, *Bifidobacterium*, *Blautia*, *Prevotella*, and *Faecalibacterium* showed significant differences among the three types of samples in the different age stages. Additionally, in the *Bifidobacterium* community, *B. adolescentis*, *B. ruminantium*, *B. longum* subsp. *infantis*, and *B. bifidum* showed different relative abundances in maternal and child feces. Meanwhile, in the *Lactobacillus* community, *L. paragasseri* showed significantly higher relative abundance in infant feces when the infant was younger than one month of age.

## Data Availability

The data presented in this study are available in Supplementary Materials here.

## Supplementary Materials

The following are available online at https://www.mdpi.com/article/10.3390/microorganisms9091995/s1, Figure S1. Alpha diversity (A) and Bifidobacterium composition at species level in maternal and infant feces (B). Figure S2. Alpha diversity (A) and *Lactobacillus* composition at species level in maternal and infant feces (B).

## References

[1] Ding M., Yang B., Ross R.P., Stanton C., Zhao J., Zhang H., Chen W. (2021). Crosstalk between sIgA-coated bacteria in infant gut and early-life health. Trends Microbiol.

[2] Gomez de Agüero M., Ganal-Vonarburg S.C., Fuhrer T., Rupp S., Uchimura Y., Li H., Steinert A., Heikenwalder M., Hapfelmeier S., Sauer U. (2016). The maternal microbiota drives early postnatal innate immune development. Science.

[3] Robertson R.C., Manges A.R., Finlay B.B., Prendergast A. (2019). The Human Microbiome and Child Growth—First 1000 Days and Beyond. Trends Microbiol..

[4] Prendergast A.J., Humphrey J.H. (2014). The stunting syndrome in developing countries. Paediatr. Int. Child Health.

[5] Martin V.N.R., Schwab C., Krych L., Voney E., Geirnaert A., Braegger C., Lacroix C. (2019). Colonization of Cutibacterium avidum during infant gut microbiota establishment. FEMS Microbiol. Ecol..

[6] Korpela K., Costea P.I., Coelho L.P., Kandels-Lewis S., Willemsen G., Boomsma D.I., Segata N., Bork P. (2018). Selective maternal seeding and environment shape the human gut microbiome. Genome Res..

[7] Wang S., Ryan C.A., Boyaval P., Dempsey E.M., Ross R., Stanton C. (2020). Maternal Vertical Transmission Affecting Early-life Microbiota Development. Trends Microbiol..

[8] Ferretti P., Pasolli E., Tett A., Asnicar F., Gorfer V., Fedi S., Armanini F., Truong D.T., Manara S., Zolfo M. (2018). Mother-to-Infant Microbial Transmission from Different Body Sites Shapes the Developing Infant Gut Microbiome. Cell Host Microbe.

[9] Pannaraj P.S., Li F., Cerini C., Bender J.M., Yang S., Rollie A., Adisetiyo H., Zabih S., Lincez P.J., Bittinger K. (2017). Association Between Breast Milk Bacterial Communities and Establishment and Development of the Infant Gut Microbiome. JAMA Pediatr..

[10] Williams J.E., Carrothers J.M., A Lackey K., Beatty N.F., Brooker S.L., Peterson H.K., Steinkamp K.M., A York M., Shafii B., Price W.J. (2019). Strong Multivariate Relations Exist Among Milk, Oral, and Fecal Microbiomes in Mother-Infant Dyads During the First Six Months Postpartum. J. Nutr..

[11] Differding M.K., Mueller N.T. (2020). Human Milk Bacteria: Seeding the Infant Gut?. Cell Host Microbe.

[12] Khine W.W.T., Rahayu E.S., See T.Y., Kuah S., Salminen S., Nakayama J., Lee Y.-K. (2020). Indonesian children fecal microbiome from birth until weaning was different from microbiomes of their mothers. Gut Microbes.

[13] Jost T., Lacroix C., Braegger C.P., Rochat F., Chassard C. (2013). Vertical mother-neonate transfer of maternal gut bacteria via breastfeeding. Environ. Microbiol..

[14] Chen Y., Yang B., Ross R.P., Jin Y., Stanton C., Zhao J., Zhang H., Chen W. (2019). Orally Administered CLA Ameliorates DSS-Induced Colitis in Mice via Intestinal Barrier Improvement, Oxidative Stress Reduction, and Inflammatory Cytokine and Gut Microbiota Modulation. J. Agric. Food Chem..

[15] Fadrosh D.W., Ma B., Gajer P., Sengamalay N., Ott S., Brotman R.M., Ravel J. (2014). An improved dual-indexing approach for multiplexed 16S rRNA gene sequencing on the Illumina MiSeq platform. Microbiome.

[16] Yang B., Ding M., Chen Y., Han F., Yang C., Zhao J., Malard P., Stanton C., Ross R.P., Zhang H. (2021). Development of gut microbiota and Bifidobacterium communities of neonates in the first 6 weeks and their inheritance from mother. Gut Microbes.

[17] Mahbubi A., Uchiyama T., Hatanaka K. (2019). Capturing consumer value and clustering customer preferences in the Indonesian halal beef market. Meat Sci..

[18] Martinez Arbizu P. (2019). pairwiseAdonis: Pairwise Multilevel Comparison Using Adonis. https://mirrors.tuna.tsinghua.edu.cn/CRAN/.

[19] Gonzalez E., Brereton N.J.B., Li C., Leyva L.L., Solomons N.W., Agellon L.B., Scott M.E., Koski K.G. (2021). Distinct Changes Occur in the Human Breast Milk Microbiome Between Early and Established Lactation in Breastfeeding Guatemalan Mothers. Front. Microbiol..

[20] Lappan R., Classon C., Kumar S., Singh O.P., de Almeida R.V., Chakravarty J., Kumari P., Kansal S., Sundar S., Blackwell J.M. (2019). Meta-taxonomic analysis of prokaryotic and eukaryotic gut flora in stool samples from visceral leishmaniasis cases and endemic controls in Bihar State India. PLoS Negl. Trop. Dis..

[21] Rodríguez J.M. (2014). The origin of human milk bacteria: Is there a bacterial entero-mammary pathway during late pregnancy and lactation?. Am. Soc. Nutr..

[22] Jost T., Lacroix C., Braegger C., Chassard C. (2013). Assessment of bacterial diversity in breast milk using culture-dependent and culture-independent approaches. Br. J. Nutr..

[23] Murphy K., Curley D., O’Callaghan T., O’Shea C.-A., Dempsey E.M., O’Toole P., Ross R., Ryan C.A., Stanton C. (2017). The Composition of Human Milk and Infant Faecal Microbiota Over the First Three Months of Life: A Pilot Study. Sci. Rep..

[24] Cabrera-Rubio R., Collado M.C., Laitinen K., Salminen S., Isolauri E., Mira A. (2012). The human milk microbiome changes over lactation and is shaped by maternal weight and mode of delivery. Am. J. Clin. Nutr..

[25] Fouhy F., Clooney A.G., Stanton C., Claesson M.J., Cotter P.D. (2016). 16S rRNA gene sequencing of mock microbial populations- impact of DNA extraction method, primer choice and sequencing platform. BMC Microbiol..

[26] Nakayama J., Watanabe K., Jiang J., Matsuda K., Chao S.-H., Haryono P., La-Ongkham O., Sarwoko M.-A., Sujaya I.N., Zhao L. (2015). Diversity in gut bacterial community of school-age children in Asia. Sci. Rep..

[27] Wu G.D., Chen J., Hoffmann C., Bittinger K., Chen Y.Y., Keilbaugh S.A., Bewtra M., Knights D., Walters W.A., Knight R. (2011). Linking Long-Term Dietary Patterns with Gut Microbial Enterotypes. Science.

[28] Princisval L., Rebelo F., Williams B.L., Coimbra A.C., Crovesy L., Ferreira A.L., Kac G. (2021). Association Between the Mode of Delivery and Infant Gut Microbiota Composition Up to six months of Age: A Systematic Literature Review Considering the Role of Breastfeeding. Nutr. Rev..

[29] Bäckhed F., Roswall J., Peng Y., Feng Q., Jia H., Kovatcheva-Datchary P., Li Y., Xia Y., Xie H., Zhong H. (2015). Dynamics and Stabilization of the Human Gut Microbiome during the First Year of Life. Cell Host Microbe.

[30] Mackie R.I., Sghir A., Gaskins H.R. (1999). Developmental microbial ecology of the neonatal gastrointestinal tract. Am. J. Clin. Nutr..

[31] Sindi A.S., Geddes D.T., E Wlodek M., Muhlhausler B.S., Payne M.S., Stinson L.F. (2021). Can we modulate the breastfed infant gut microbiota through maternal diet?. FEMS Microbiol. Rev..

[32] Fernández L., Langa S., Martin V., Maldonado A., Jiménez E., Martín R., Rodríguez J.M. (2013). The human milk microbiota: Origin and potential roles in health and disease. Pharmacol. Res..

[33] He X., Parenti M., Grip T., Domellöf M., Lönnerdal B., Hernell O., Timby N., Slupsky C.M. (2019). Metabolic phenotype of breast-fed infants, and infants fed standard formula or bovine MFGM supplemented formula: A randomized controlled trial. Sci. Rep..

[34] Fitzstevens J.L., Smith K.C., Hagadorn J.I., Caimano M.J., Matson A.P., Brownell E.A. (2016). Systematic Review of the Human Milk Microbiota. Nutr. Clin. Pract..

[35] Treven P., Mahnič A., Rupnik M., Golob M., Pirš T., Matijašić B.B., Lorbeg P.M. (2019). Evaluation of human milk microbiota by 16S rRNA gene next-generation sequencing (NGS) and sultivation/MALDI-TOF mass spectrometry identification. Front. Microbiol..

[36] Wessels J.M., Lajoie J., Cooper M.I.H., Omollo K., Felker A.M., Vitali D., Haley A., Dupont P.V.N. (2019). Medroxyprogesterone acetate alters the vaginal microbiota and microenvironment in women and increases susceptibility to HIV-1 in humanized mice. Dis. Model Mech..

[37] Yang B., Yan S., Chen Y., Ross R.P., Stanton C., Zhao J., Zhang H., Chen W. (2020). Diversity of gut microbiota and Bifidobacterium community of Chinese subjects of different ages and from different regions. Microorganisms.

[38] Li T., Yang J., Zhang H., Xie Y., Jin J. (2020). Bifidobacterium from breastfed infant faeces prevent high-fat-diet-induced glucose tolerance impairment, mediated by the modulation of glucose intake and the incretin hormone secretion axis. J. Sci. Food Agric..

[39] Moya-Perez A., Perez-Villalba A., Benitez-Paez A., Campillo I., Sanz Y. (2017). Bifidobacterium CECT 7765 modulates early stress-induced immune, neuroendocrine and behavioral alterations in mice. Brain Behav. Immun..

[40] Nakayama J., Yamamoto A., Palermo-Conde L.A., Higashi K., Sonomoto K., Tan J., Lee Y.-K. (2017). Impact of Westernized Diet on Gut Microbiota in Children on Leyte Island. Front. Microbiol..

[41] Hill C.J., Lynch D.B., Murphy K., Ulaszewska M., Jeffery I.B., O’Shea C.A., Watkins C., Dempsey E., Mattivi F., Tuohy K. (2017). Evolution of gut microbiota composition from birth to 24 weeks in the INFANTMET Cohort. Microbiome.

[42] Fouhy F., Watkins C., Hill C.J., O’Shea C.A., Nagle B., Dempsey E.M., Stanton C. (2019). Perinatal factors affect the gut microbiota up to four years after birth. Nat. Commun..

[43] Dzidic M., Boix-Amorós A., Selma-Royo M., Mira A., Collado M.C. (2018). Gut Microbiota and Mucosal Immunity in the Neonate. Med. Sci..

[44] Kim Y.-G., Sakamoto K., Seo S.-U., Pickard J.M., Gillilland M.G., Pudlo N.A., Hoostal M., Li X., Wang T.D., Feehley T. (2017). Neonatal acquisition of Clostridia species protects against colonization by bacterial pathogens. Science.

[45] Wang S., Hibberd M.L., Pettersson S., Lee Y.K. (2014). Enterococcus faecalis from Healthy Infants Modulates Inflammation through MAPK Signaling Pathways. PLoS ONE.

[46] Yang B., Chen Y., Stanton C., Ross R.P., Lee Y.-K., Zhao J., Zhang H., Chen W. (2019). Bifidobacterium and Lactobacillus Composition at Species Level and Gut Microbiota Diversity in Infants before 6 Weeks. Int. J. Mol. Sci..

[47] Zhang Q., Hu J., Feng J., Hu X.-T., Wang T., Gong W.-X., Huang K., Guo Y.-X., Zou Z., Lin X. (2020). Influenza infection elicits an expansion of gut population of endogenous Bifidobacterium animalis which protects mice against infection. Genome Biol..

[48] Klaassens E.S., Boesten R.J., Haarman M., Knol J., Schuren F.H., Vaughan E.E., de Vos W.M. (2009). Mixed-Species Genomic Microarray Analysis of Fecal Samples Reveals Differential Transcriptional Responses of Bifidobacteria in Breast- and Formula-Fed Infants. Appl. Environ. Microbiol..

[49] Duranti S., Turroni F., Lugli G.A., Milani C., Viappiani A., Mangifesta M., Gioiosa L., Palanza P., van Sinderen D., Ventura M. (2014). Genomic Characterization and Transcriptional Studies of the Starch-Utilizing Strain Bifidobacterium adolescentis 22L. Appl. Environ. Microbiol..

[50] Gavini F., Delcenserie V., Kopeinig K., Pollinger S., Beerens H., Bonaparte C., Upmann M. (2006). Bifidobacterium species isolated from animal two and from beef and pork meat. J. Food Prot..

[51] James K., Motherway M.O., Bottacini F., Van Sinderen D. (2016). Bifidobacterium breve UCC2003 metabolises the human milk oligosaccharides lacto-N-tetraose and lacto-N-neo-tetraose through overlapping, yet distinct pathways. Sci. Rep..

[52] Turroni F., Ventura M., Buttó L.F., Duranti S., O’Toole P., Motherway M.O., Van Sinderen D. (2014). Molecular dialogue between the human gut microbiota and the host: A Lactobacillus and Bifidobacterium perspective. Cell. Mol. Life Sci..

[53] Zhang X., Mushajiang S., Luo B., Tian F., Ni Y., Yan W. (2020). The Composition and Concordance of Lactobacillus Populations of Infant Gut and the Corresponding Breast-Milk and Maternal Gut. Front. Microbiol..

[54] Petrova M.I., Lievens E., Malik S., Imholz N., Lebeer S. (2015). Lactobacillus species as biomarkers and agents that can promote various aspects of vaginal health. Front. Physiol..

[55] Mortensen M.S., Rasmussen M.A., Stokholm J., Brejnrod A.D., Balle C., Thorsen J., Krogfelt K.A., Bisgaard H., Sørensen S.J. (2021). Modeling transfer of vaginal microbiota from mother to infant in early life. eLife.

[56] Lai H.-H., Chiu C.-H., Kong M.-S., Chang C.-J., Chen C.-C. (2019). Probiotic Lactobacillus casei: Effective for Managing Childhood Diarrhea by Altering Gut Microbiota and Attenuating Fecal Inflammatory Markers. Nutrients.

[57] Qi C., Ding M., Li S., Zhou Q., Li D., Yu R., Sun J. (2021). Sex-dependent modulation of immune development by sIgA coated Lactobacillus reuteri isolated from breast milk. J. Dairy Sci..

