# Supplementary material for: The Species-Level Composition of the Fecal Bifidobacterium and Lactobacillus Genera in Indonesian Children Differs from That of Their Mothers
Source: Microorganisms. 2021 Sep 21;9(9):1995. doi: 10.3390/microorganisms9091995 (PMC8467263; doi:10.3390/microorganisms9091995)

Figure S1. Alpha-diversity illustrating richness and diversity in *Bifidobacterium* community (A) and *Bifidobacterium* composition at species level in maternal and infant feces (B).

MFA: maternal feces (corresponding to infant younger than 1 month of age), MFB: maternal feces (corresponding to infant between 1 and 3 months of age), MFC: maternal feces (corresponding to infant between 3 and 6 months of age), MFD: maternal feces (corresponding to infant between 6 and 12 months of age)，MFE: maternal feces (corresponding to infant older than 12 months of age), BFA: infant feces younger than 1 month of age, BFB: infant feces between 1 and 3 months of age, BFC: infant feces between 3 and 6 months of age, BFD: infant feces between 6 and 12 months of age，BFE: infant feces older than 12 months of age.

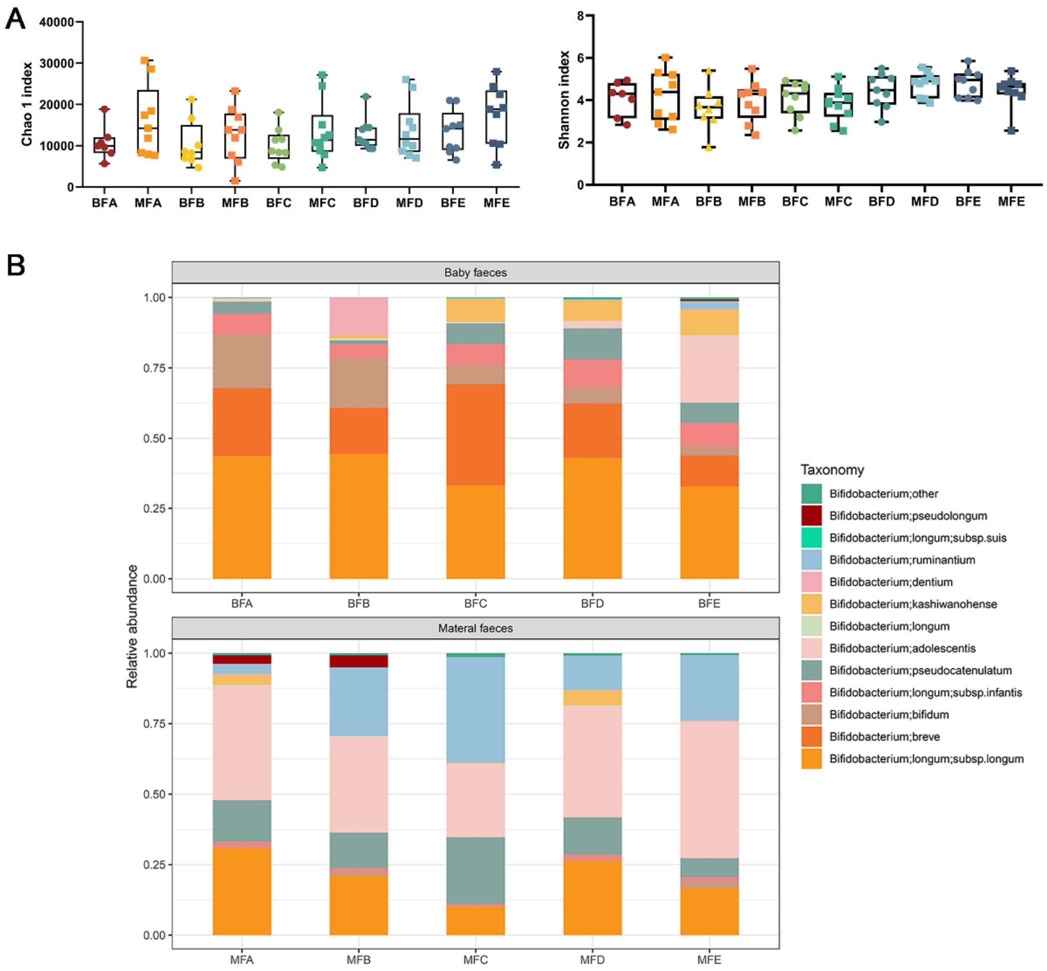

Figure S2. Alpha-diversity illustrating richness and diversity in *Lactobacillus* community (A) and *Lactobacillus* composition at species level in maternal and infant feces (B).

*, $p < 0.05$; MFA: maternal feces (corresponding to infant younger than 1 month of age), MFB: maternal feces (corresponding to infant between 1 and 3 months of age), MFC: maternal feces (corresponding to infant between 3 and 6 months of age), MFD: maternal feces (corresponding to infant between 6 and 12 months of age)，MFE: maternal feces (corresponding to infant older than 12 months of age), BFA: infant feces younger than 1 month of age, BFB: infant feces between 1 and 3 months of age, BFC: infant feces between 3 and 6 months of age, BFD: infant feces between 6 and 12 months of age，BFE: infant feces older than 12 months of age.

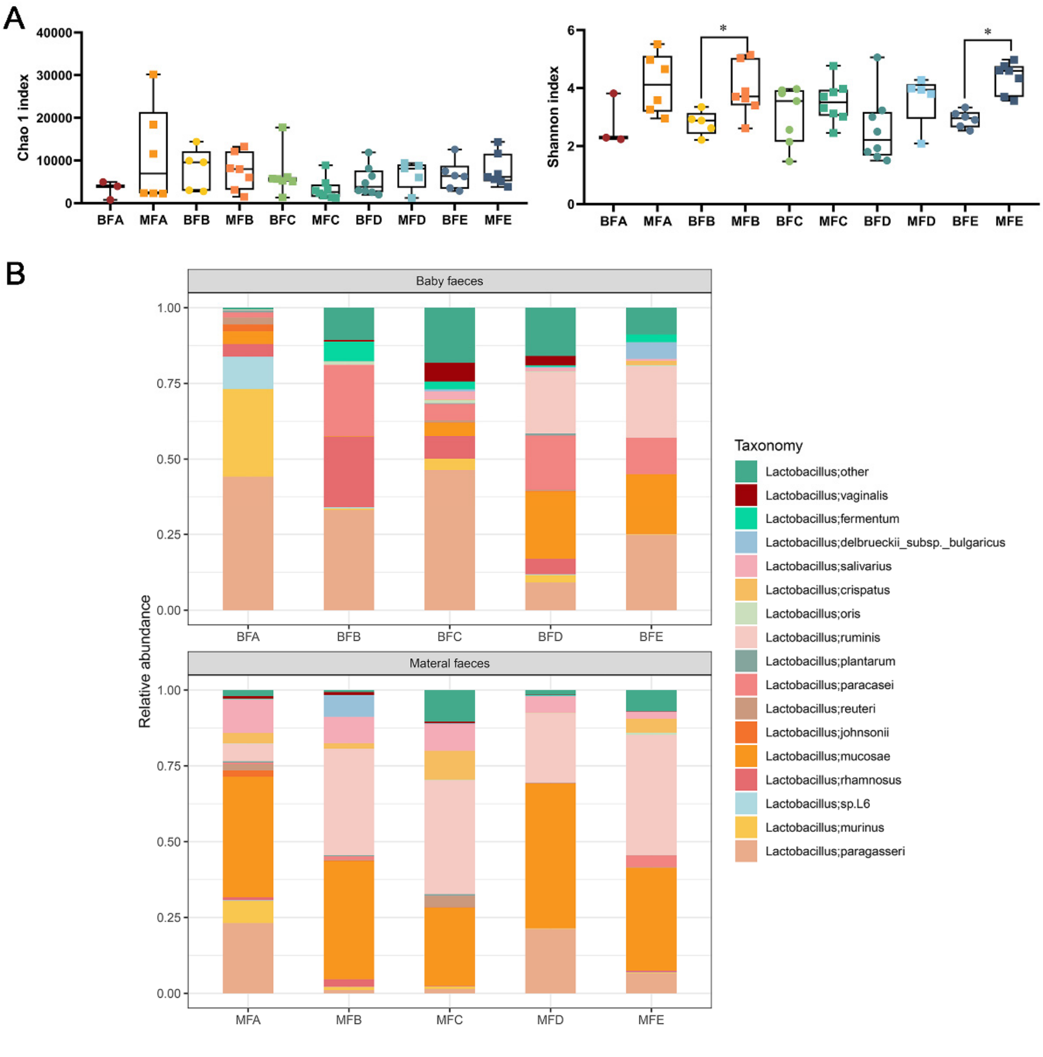

Supplement: Supplementary file 1 [file microorganisms-09-01995-s001.zip › microorganisms-1376697-supplementary.pdf]
